# Evaluation of 12-Lipoxygenase (12-LOX) and Plasminogen Activator Inhibitor 1 (PAI-1) as Prognostic Markers in Prostate Cancer

**DOI:** 10.1155/2014/102478

**Published:** 2014-03-24

**Authors:** Tomasz Gondek, Mariusz Szajewski, Jarosław Szefel, Ewa Aleksandrowicz-Wrona, Ewa Skrzypczak-Jankun, Jerzy Jankun, Wieslawa Lysiak-Szydlowska

**Affiliations:** ^1^Department of Urology, St' Vincent A Paulo Hospital, Wójta Radtkego 1, 81-348 Gdynia, Poland; ^2^Department of Urology, Multidisciplinary Hospital Jantar, Rybacka 15, 82-103 Jantar, Poland; ^3^Department of Surgical Oncology, Gdynia Oncology Center, PCK's Maritime Hospital in Gdynia, Powstania Styczniowego 1, 81-519 Gdynia, Poland; ^4^Department of Propaedeutic Oncology, Faculty of Health Sciences, Medical University of Gdańsk, Powstania Styczniowego 9b, 81-519 Gdynia, Poland; ^5^Department of Clinical Nutrition, Medical University of Gdańsk, Dębinki 7, 80-211 Gdańsk, Poland; ^6^Urology Research Center, Department of Urology, The University of Toledo, Health Science Campus, Toledo, OH 43614, USA; ^7^Protein Research Chair, Department of Biochemistry, College of Sciences, King Saud University, Riyadh 11451, Saudi Arabia; ^8^Powiślanski College, Faculty of Health Science, 11 Listopada 13, 82-500 Kwidzyń, Poland

## Abstract

In carcinoma of prostate, a causative role of platelet 12-lipoxygenase (12-LOX) and plasminogen activator inhibitor 1 (PAI-1) for tumor progression has been firmly established in tumor and/or adjacent tissue. Our goal was to investigate if 12-LOX and/or PAI-1 in patient's plasma could be used to predict outcome of the disease. The study comprised 149 patients (age 70 ± 9) divided into two groups: a study group with carcinoma confirmed by positive biopsy of prostate (*n* = 116) and a reference group (*n* = 33) with benign prostatic hyperplasia (BPH). The following parameters were determined by the laboratory test in plasma or platelet-rich plasma: protein level of 12-LOX, PAI-1, thromboglobulin (TGB), prostate specific antigen (PSA), C-reactive protein (CRP), hemoglobin (HGB, and hematocrit (HCT), as well as red (RBC) and white blood cells (WBC), number of platelets (PLT), international normalized ratio of blood clotting (INR), and activated partial thromboplastin time (APTT). The only difference of significance was noticed in the concentration of 12-LOX in platelet rich plasma, which was lower in cancer than in BPH group. Standardization to TGB and platelet count increases the sensitivity of the test that might be used as a biomarker to assess risk for prostate cancer in periodically monitored patients.

## 1. Introduction

The prostate cancer is the most common malignancy diagnosed in older men in the Western Hemisphere population. According to the European Association of Urology (EAU) Guidelines of 2012 mortality from prostate cancer is ranked second to lung cancer [1]. The diagnosis of prostate cancer at an early stage and ability to differentiate benign and aggressive form would improve the selection of the optimal method of treatment resulting in better outcome. Currently used diagnostic standards consist of determination of prostate specific antigen (PSA), clinical stage, and total Gleason grade. Unfortunately, they do not give sufficient justification for choosing the optimal therapy for a particular patient. Hence, it is necessary to search for new biomarkers to allow for the prediction of disease dynamics and personalization of therapy [2, 3].

It has been shown that men who consume high-fat diet containing abundance of arachidonic acid (AA) have a high rate of incidence of prostate cancer [4, 5]. Availability of AA in combination with the overexpression of lipoxygenases (12-LOX, 5-LOX), cyclooxygenase (COX-2), and cytochrome P450 (CYP) excess leads to the synthesis of eicosanoids [6–8]. Eicosanoids trigger signals for transcription of genes that modulate the immune system, hemostasis, apoptosis, cell proliferation, and many other processes [9, 10]. This avalanche of signals results in the development of inflammation favoring carcinogenesis [11, 12]. Also, eicosanoids accelerate the rate of proliferation of glandular cells, inhibit their apoptosis, and further intensify angiogenesis [13–16]. Angiogenesis is a prerequisite for tumor development and plasminogen activation system (PAS) has a significant impact on that crucial step. PAS includes urokinase plasminogen activator (uPA), urokinase plasminogen activator receptor (uPAR), and plasminogen activator inhibitor type-1 (PAI-1) [17]. The increase in uPA activity and number of uPAR correlate with the ability of cancer to form angiogenic vasculature and increase cancer cells metastasis. Urokinase, both free and receptor bound, converts plasminogen to proteolytically active plasmin that is responsible for lysis of extracellular matrix, essential for angiogenesis and metastasis [18, 19]. Inhibition of both uPA and uPAR activity reduces angiogenesis and metastasis [20, 21]. Research indicates that inhibition of uPA by PAI-1 reduces the size of the tumor [22]. In the capillaries surrounding the tumor there are a large amount and activity of uPA and uPAR [23]. Taking into account the role of uPA, uPAR, and PAI-1 in angiogenesis, Pepper considers that normal vessel formation by angioge-nesis depends on proteases and antiproteases balance [24]. However, role of PAI-1 in carcinogenesis is more complex than simple inhibition of proteolysis. PAI-1 overexpressed up to approximately ten times more than normal level increases motility of cancer cells by interacting with vitronectin and other proteins. However, PAI-1 in supramolecular levels significantly inhibits angiogenesis and metastasis reducing activity of uPA [18, 25, 26]. This phenomenon is called “PAI-paradox” [27]. Now, a high level of PAI-1 appears to inhibit angiogenesis, while slightly elevated level of PAI-1 is necessary for growth of angiogenic vessels.

A healthy body maintains a balance between activators and inhibitors of angiogenesis. The tumor microenvironment is different than normal tissue where the pro- and antiangiogenic factors are well balanced. Folkman and Hanahan introduced the concept of the angiogenic switch wherein it is stated that angiogenesis starts at a global disturbance of the expression of pro- and antiangiogenic factors [28]. The primary target of both of them is endothelial cell [29]. Among the others 12-LOX and PAI-1 are proteins governing these processes and can be secreted at high levels by tumor cells. While expression of these proteins by cancer cell was studied and documented, serum tests were not well investigated [30–32], albeit they might provide an easy laboratory test as a diagnostic tool. Therefore, we have studied expression of human platelet 12-LOX and PAI-1 in serum of patients to find whether any correlation exists between their concentration in blood and stage of the prostate disease.

## 2. Materials and Methods

### 2.1. Patients

The study involved 149 men (age 70 ± 9 years) qualified for diagnostic biopsy of the prostate. The criteria for inclusion in the study were a positive digital rectal examination (DRE) result, PSA level above the upper limit of the reference value of 4 ng/mL, and a positive transrectal ultrasound (TRUS) test result. The study excluded patients with previously diagnosed cancer, regardless of its location and nature. Patients taking aspirin, warfarin, COX inhibitors, and heparin were also excluded from study since these drugs may affect level or activity of 12-LOX and/or PAI-1 [33–37].

In all patients the entire volume of the prostate adenoma and cancer foci were determined by TRUS. Biopsy was done for all patients with a total of 12 samples taken in the following way: biopsies 1–4 taken from the suspected foci, biopsies 5–8 taken from the opposite lobe of the prostate, and biopsies 9–12 taken from the lobe where the suspected foci were present. Targeted biopsy was performed on patients suspected with pathological growth. Formaldehyde and paraffin embedded slides were examined by pathologist who determined the type of cancer (or lack of it), tumor grade and Gleason sum. Based on the results of the histopathological examination of biopsy material the patients were divided into two groups (group with cancer, *n* = 116, and without prostate cancer, *n* = 33). The study was approved by the Bioethical Committee for Scientific Research at the Medical University of Gdańsk. Each patient prior to study was informed of the objectives and principles and signed an informed consent to participate in it.

### 2.2. Blood

For all patients the following parameters were determined in hospital laboratory by the routine tests: hemoglobin (HGB), hematocrit (HCT), red blood cells (RBC), and white blood cells (WBC). Citrated blood samples were divided into two parts, one of them was centrifuged at 100 ×g for fifteen minutes to obtain platelet rich plasma, frozen, and stored at −20°C for the determination of PAI-1, 12-LOX, and thromboglobulin (TGB); the other was centrifuged at 1500×g for ten minutes for the determination of PSA, C-reactive protein (CRP), international normalized ratio of blood clotting (INR), and activated partial thromboplastin time (APTT).

### 2.3. ELISA Kits

Thromboglobulin was assayed by Asserachrom-TBG, product number REF 00950 from Diagnostic Stago Inc., Mount Olive, NJ, USA. PAI-1 was analyzed by active human PAI-1 functional assay ELISA kit, HPAIKIT from Molecular Innovation, Novi, MI 48377, USA. 12-LOX was analyzed by IMUBIND 12-Lipoxygenase ELISA product number ADG872 from American Diagnostica GmbH, Pfungstadt, Germany.

### 2.4. Statistical Analysis

Statistical analysis was done using Statistica 10 (StatSoft Polska Sp. z o.o., Krakόw, Poland) for nonparametric Mann-Whitney *U* test, Chi square distribution, and analysis of the Pearson correlation coefficient. The level of significance was established as *P* ≤ 0.05.

## 3. Results and Discussion

Enrolled patients in addition to BPH and prostate cancer were diagnosed with diabetes (12%), hypertension (42%), chronic obstructive pulmonary disease (6%), coronary disease (94%), diabetes (10.3%), and hypertension (50.8%). Patients in study were divided into two groups: with prostate cancer and with benign prostatic hyperplasia (BPH). No prevalence of any of these diseases was observed between BPH and prostate cancer patients. Blood work revealed also that there were no differences in test values of CRP, APTT, HGB, HCT, WBC, RBC, PLT, and TGB between these two groups (data not shown). As can be seen in Table 1 patients with BPH were younger and have had higher volume of prostate, volume of adenoma and, as expected, significantly lower PSA than these with prostate cancer.

**Figure 1:**
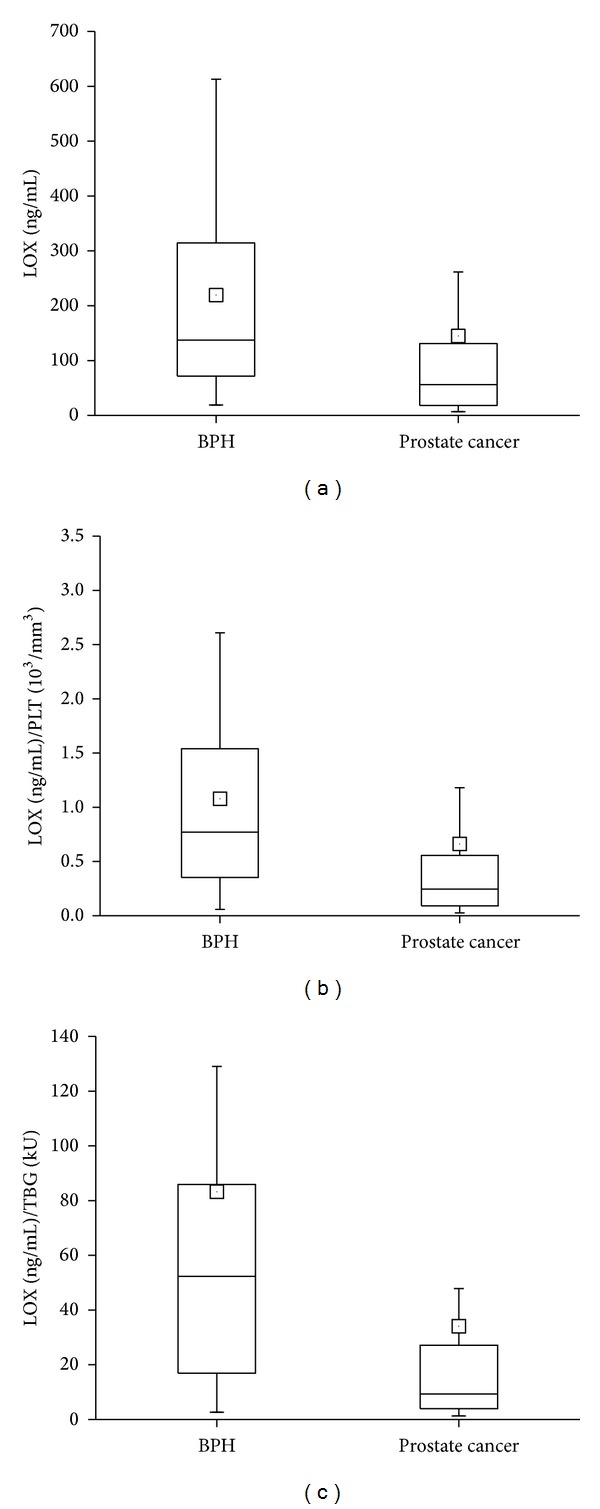
Expression of 12-LOX in platelet-rich plasma of BPH and prostate cancer patients. Normalization to PLT and TBG greatly increases sensitivity of correlation. Box and whisker plots of expression of 12-LOX (*P* = 0.0001) (a), 12-LOX normalized to PLT (*P* = 0.00003) (b), and 12-LOX normalized to TBG (*P* = 0.000005) (c) for BPH and prostate cancer. Solid, horizontal line inside box represents the median, position of the little square gives the average, box encompasses results within 25–75%, and wiskers mark values between 5–95%.

Table 2 shows that expression of 12-LOX in platelet rich plasma was significantly lower in prostate cancer patients than in BPH population and normalization to PLT and TBG increases statistical significance (Figure 1). Differences between the groups in all other tested parameters were not statistically significant.

Also as it is shown in Table 3 there were no differences in tested parameters in prostate cancer patients divided for groups according to the Gleason grade <6 and >6, respectively.

This study includes only BPH and prostate cancer patients. Healthy individuals were excluded due to an ethical consideration. Defining a person as “healthy” would require verification by blood work, digital rectum examination, and biopsy which (especially biopsy) was considered unethical for the asymptomatic person.

Cancer markers indicate a high probability for the existence of cancer in the body. Most markers are assayed by analysis of blood plasma [38]. It is expected that the concentration of tumor markers in the plasma or urine of patients with cancer should considerably vary from the values typically observed in healthy subjects [39]. This assumption results from the positive relationship between the mass of cancer cells and the amount of the substance produced by them [40]. At this moment markers of prostate cancer cannot select precisely a group at risk for the disease progression [41, 42]. The 12-LOX and PAI-1 together with the products of the reactions catalyzed by them seem to point an interesting direction of research [30, 43]. In our initial studies we measured 12-LOX expression in serum and found that 12-LOX was lower in prostate cancer patients in comparison with healthy individuals and BPH patients. However limited number of individuals in each group did not allow us to establish statistical significant differences [44]. The other studies show promise but are difficult to compare due to the fact that some of them analyzed prostate tissue, while other were done in plasma [26, 45, 46]. Also, one study analyzed the expression of genes; the other reported levels of protein, and yet in some other the enzyme activity in plasma was determined for 12-LOX and PAI-1 [47, 48]. The disparities were also observed in the method of selecting groups of patients. Studies compared the results for patients without and with cancer, which could mean healthy, but also from cobenign prostatic hyperplasia [8, 14, 49, 50]. Moreover, laboratory tests for the determination of 12-LOX and PAI-1 used antibodies of different specificity. These diversities make the comparison of the results difficult and rather questionable.

Higher PSA depending on the severity of cancer, lower volume of cancerous prostate confirm not only the generally accepted and recognized standards of diagnosis and management in the field of prostate cancer, but also the proper selection of patients in the study group. To improve the results in our study we normalized assayed parameters to the number of platelets and TBG in platelet rich plasma. Each platelet rich plasma sample was frozen for storage (not exceeding 12–16 weeks) and thaw immediately before the appropriate tests were performed to guarantee uniform conditions for releasing TBG and other proteins in the determination of platelet-rich plasma parameters studied [51].

Analyzing the results for the concentrations of TBG in the platelet-rich plasma, we have observed that the differences in the TBG levels of *P* = 0.054 (Table 2) where close to the chosen level of significance *P* less or equal 0.05, with the mean value of TBG higher in the cancer group. This somewhat diminished that statistical level of significance may be due to the fact that the control group consisted of patients with BPH and not prostate-trouble-free individuals.

The activity of the platelet 12-LOX in prostate cancer was investigated extensively and tied with angiogenesis. Nie et al. examined 12-LOX concentration in prostate cancer cell lines using antibodies and postulated that increase of 12-LOX expression stimulates prostate cancer tumor growth and activates angiogenesis [13]. Elevated levels or activity in cancer tissue has been reported before by others as well [30, 52–55]; thus, our finding of reduced amount of 12-LOX in platelet-rich plasma of prostate cancer patients was somewhat surprising. One of the explanations is that volume of prostate in BPH patients was larger than prostate cancer patients. So, if BPH and prostate cancer gland release steady amount of 12-LOX into blood, indeed this protein level can depend on gland volume. It is worthy to emphasize that a mean total volume of prostate in cancer patients was 65% of BPH prostate group of patients, and percentage of 12-LOX expression in platelet rich plasma was also 65% for cancer group versus patients with BPH. Moreover, the mean volume of cancer foci was only ~1% of prostate volume in prostate cancer patients so its impact on total secretion of 12-LOX in blood could be limited. The other possibility is that lower 12-LOX expression in cancer is intrinsic property of cancer. Observations from the cell lines are consistent with the results from human tissue in the fact that 12-LOX level is growing with the progression of cancer. However, Gohara et al. reported that expression of 12-LOX in normal kidney tissue is higher than in low grade and stage form of this cancer, to rise in terminal malignancy, approximating but not quite reaching the level of expression observed in the normal tissue samples [56]. Thus this mechanism requires more investigations.

Expression of 12-LOX also depends on type of cancer. For example it has been reported that significant increase in 12-LOX levels in serum was observed in breast cancer patients (40 ng/mL) as compared to healthy controls (13 ng/mL) (*P* < 0.0001) in study of 86 biopsy proven breast cancer patients. Moreover, serum 12-LOX levels were significantly higher (*P* < 0.002) in patients with metastasis to the lymph nodes and over 75% patients had shown significant (*P* < 0.0001) reduction of 12-LOX levels after chemotherapy [57].

We have not seen any significant differences in expression of PAI-1 in platelet rich plasma between BPH and prostate cancer patients, as well as in groups with different Gleason grade (Table 3), regardless of many reports stating that PAI-1 is overexpressed in prostate cancer [43, 58, 59], although uPA and its receptor are overexpressed on the surface of cancer cells. However, when PAI-1 binds to uPA-uPAR complexes it interacts with LPR leading to internalization of PAI-1/uPA-uPAR/LPR into the cancer cells. PAI-1 and uPA are degraded while uPAR and LPR are recycled to the cell surface [25, 60–63]. Thus PAI-1 might not be secreted into the blood stream. Although expression of 12-LOX and PAI-1 and normalized expression to TBG and PLT in prostate cancer patients clearly were not statistically different in patients with different Gleason grade (Table 3), but some trend was observed. Concentrations of 12-LOX were lower in group with Gleason >6, while PAI-1 was higher when compared with group of Gleason <6. Together with other parameters the Gleason grading system helps evaluate the prognosis of men with prostate cancer. Cancers with the higher Gleason score are more aggressive and have a worse prognosis but this score itself cannot predict outcome precisely [64]. Thus, it is possible that expressions of 12-LOX and PAI-1 are related to other parameters such as outcome of disease or survival which we are going to monitor in the future studies.

## 4. Conclusion

No significant difference in platelet-rich plasma was noted for PAI-1 levels or 12-LOX and PAI-1 ratio between patients with cancer and BPH. Therefore, PAI-1 results in this study do not meet the conditions expected for the prostate cancer marker.

The concentration of 12-LOX in platelet-rich plasma in patients with prostate cancer is significantly lower than in patients with BPH; thus, the low concentration of 12-LOX might indicate the increased risk of developing prostate cancer or the onset of the disease in periodically monitored patients. Standardization of the expression of 12-LOX in platelet-rich plasma to the concentration of TGB and the number of PLT significantly increases the sensitivity of the test and could be used as biomarker for the assessment of risk for the prostate cancer.

## Figures and Tables

**Table 1:** Characteristics of the study group.

	BPH Mean ± SD (median)	Prostate cancer Mean ± SD (median)	*P*
Age of patients (years)	67.3 ± 9.9 (65)	71.2 ± 8.5 (72)	0.02
Volume of prostate (mL)	64.4 ± 32.5 (55.7)	50.1 ± 24.4 (47.7)	0.01
Volume of adenoma (mL)	31.1 ± 20.8 (25.1)	22 ± 15.8 (18.3)	0.004
Volume of cancer foci (mL)	—	0.5 ± 0.9 (0.2)	
PSA (ng/mL)	6.6 ± 4.8 (5.0)	58.4 ± 302.0 (8.6)	0.0004
Gleason grade ≤ 6	—	63%	
Gleason grade > 6	—	37%	

**Table 2:** Normal expression of 12-LOX and PAI-1 and normalized expression to TBG and PLT in BPH and prostate cancer patients.

	BPH Mean ± SD (median)	Prostate cancer Mean ± SD (median)	*P*
12-LOX (ng/mL)	219.6 ± 209.3 (137)	144.6 ± 304.8 (56)	0.0001
PAI-1 (U/mL)	447.0 ± 345.8 (367)	610.8 ± 483.9 (441)	0.1
TBG (kU)	5.81 ± 6.02 (2.8)	6.21 ± 4.03 (6.7)	0.054
PLT (10^3^/mm^3^)	207 ± 55 (208)	219 ± 69 (211)	0.6
12-LOX/TBG	83.2 ± 111.2 (52.3)	34.1 ± 77.9 (9.3)	0.000005
PAI-1/TBG	217.5 ± 321.6 (120)	151.7 ± 180.9 (90)	0.2
TBG/PLT	0.02 ± 0.02 (0.01)	0.03 ± 0.02 (0.02)	0.13
12-LOX/PLT	1.07 ± 0.97 (0.8)	0.66 ± 1.40 (0.25)	0.00003
PAI-1/PLT	2.28 ± 1.68 (1.7)	2.98 ± 2.57 (2.2)	0.2

**Table 3:** Normal expression of 12-LOX and PAI-1 and normalized expression to TBG and PLT in prostate cancer patients with different Gleason grade.

	Gleason grade ≤ 6 Mean ± SD (median)	Gleason grade > 6 Mean ± SD (median)	*P*
12-LOX (ng/mL)	158.5 ± 354.0 (56)	112.8 ± 133.7 (63)	0.8
PAI-1 (U/mL)	577.3 ± 442.2 (428)	687.3 ± 568.6 (499)	0.3
TBG (kU)	6.5 ± 4.5 (7.75)	5.5 ± 2.7 (5.4)	0.4
PLT (10^3^/mm^3^)	209.9 ± 49.9 (210)	241.3 ± 97.1 (219)	0.2
12-LOX/TBG	35.6 ± 80.8 (8.8)	30.7 ± 71.7 (11.4)	0.5
PAI-1/TBG	135.8 ± 155.3 (80)	188.6 ± 227.9 (90)	0.6
TBG/PLT	0.03 ± 0.02 (0.03)	0.025 ± 0.01 (0.02)	0.3
12-LOX/PLT	0.75 ± 1.6 (0.25)	0.47 ± 0.53 (0.27)	0.9
PAI-1/PLT	2.95 ± 2.6 (2.1)	3.0 ± 2.6 (2.4)	0.7
